# Avelumab maintenance therapy for advanced urothelial carcinoma: subgroup analyses by characteristics of first-line platinum-based chemotherapy from a real-world chart review study in Japan (JAVEMACS)

**DOI:** 10.1186/s12885-026-15781-1

**Published:** 2026-03-26

**Authors:** Yuki Endo, Hiroshi Kitamura, Suguru Shirotake, Masaomi Ikeda, Kan Yonemori, Norihiko Kawamura, Atsuko Fujihara, Takashige Abe, Fumitaka Shimizu, Kiyohide Fujimoto, Tohru Nakagawa, Shingo Hatakeyama, Kiyoaki Nishihara, Daiki Ikarashi, Naoya Masumori, Sei Naito, Kazutoshi Fujita, Takuto Hara, Noriyoshi Miura, Takahito Negishi, Junji Yatsuda, Mizuki Kobayashi, Rikiya Taoka, Junya Furukawa, Michihiro Shono, Takashi Kobayashi, Eiji Kikuchi

**Affiliations:** 1 Department of Urology, Nippon Medical School Hospital, 1-1-5 Sendagi, Bunkyo-Ku, Tokyo, 113-8603 Japan; 2 Department of Urology, Faculty of Medicine, University of Toyama, Toyama, Toyama Japan; 3 Department of Uro-Oncology, Saitama Medical University International Medical Center, Hidaka, Saitama Japan; 4 Department of Urology, Kitasato University School of Medicine, Sagamihara, Kanagawa Japan; 5 Department of Medical Oncology, National Cancer Center Hospital, Chuo-Ku,Tokyo, Japan; 6 Department of Urology, Osaka International Cancer Institute, Chuo-Ku, Osaka, Japan; 7 Department of Urology, Kyoto Prefectural University of Medicine, Kamigyo-Ku, Kyoto, Japan; 8 Department of Renal and Genitourinary Surgery, Hokkaido University Graduate School of Medicine, Sapporo, Hokkaido Japan; 9 Department of Urology, Juntendo University Graduate School of Medicine, Bunkyo-Ku, Tokyo, Japan; 10 Juntendo University Shizuoka Hospital, Izunokuni, Shizuoka Japan; 11 Department of Urology, Nara Medical University, Kashihara, Nara, Japan; 12 Department of Urology, Teikyo University School of Medicine, Itabashi-Ku, Tokyo, Japan; 13 Department of Urology, Hirosaki University Graduate School of Medicine, Hirosaki, Aomori, Japan; 14 Department of Urology, Kurume University School of Medicine, Kurume, Fukuoka Japan; 15 Department of Urology, Iwate Medical University School of Medicine, Shiwa-Gun, Iwate, Japan; 16 Department of Urology, Sapporo Medical University School of Medicine, Sapporo, Hokkaido Japan; 17 Department of Urology, Yamagata University Faculty of Medicine, Yamagata, Japan; 18 Department of Urology, Kindai University Faculty of Medicine, Osakasayama, Osaka Japan; 19 Division of Urology, Kobe University Graduate School of Medicine, Kobe, Hyogo Japan; 20 Department of Urology, Ehime University Graduate School of Medicine, Toon, Ehime, Japan; 21 Department of Urology, National Hospital Organization Kyushu Cancer Center, Fukuoka, Fukuoka Japan; 22 Department of Urology, Graduate School of Medical Sciences, Kumamoto University, Kumamoto, Kumamoto Japan; 23 Department of Urology, Akita University Graduate School of Medicine, Akita, Japan; 24 Department of Urology, Faculty of Medicine, Kagawa University, Kita-gun, Kagawa, Japan; 25 Department of Urology, Tokushima University Graduate School of Biomedical Sciences, Tokushima, Tokushima, Japan; 26 Medical Department, Merck Biopharma Co., Ltd. (an affiliate of Merck KGaA), Minato-Ku, Tokyo Japan; 27 Department of Urology, Kyoto University Graduate School of Medicine, Sakyo-Ku, Kyoto, Japan; 28 Department of Urology, St Marianna University School of Medicine, Kawasaki, Kanagawa Japan

**Keywords:** Avelumab, Bladder cancer, Maintenance, Platinum-based chemotherapy, Real-world study, Retrospective, Urothelial carcinoma

## Abstract

**Background:**

Avelumab maintenance therapy was approved in Japan in February 2021 for patients with unresectable locally advanced or metastatic urothelial carcinoma (la/mUC) without disease progression after platinum-based chemotherapy (PBC). We report subgroup analyses from the JAVEMACS chart review study of avelumab maintenance therapy in Japan by characteristics of first-line (1L) PBC.

**Methods:**

This multicenter, retrospective study collected data from medical charts of patients with la/mUC who received avelumab maintenance after 1L PBC. Protocol-specified subgroups defined by 1L PBC characteristics (PBC regimen, number of PBC cycles, and best overall response to PBC) were analyzed.

**Results:**

Between February 2021 and December 2023, 350 patients received avelumab maintenance. Patient characteristics were generally similar across subgroups defined by 1L PBC characteristics. In patients who received 1L gemcitabine + cisplatin (*n* = 196) or gemcitabine + carboplatin (*n* = 116), median overall survival (OS) was not reached (95% CI, 31.2 months-not estimable [NE]) and 24.3 months (95% CI, 19.5–30.6 months), respectively. In patients who received 1–3 cycles (*n* = 68), 4 cycles (*n* = 205), 5/6 cycles (*n* = 61), or ≥ 7 cycles (*n* = 16) of 1L PBC, median OS was 25.0 months (95% CI, 15.2 months-NE), not reached (95% CI, 29.3 months-NE), 31.2 months (95% CI, 18.0 months-NE), and 23.6 months (95% CI, 19.3 months-NE), respectively. In patients with complete response (*n* = 32), partial response (*n* = 180), or stable disease (*n* = 138) as best response to 1L PBC, median OS was not reached (95% CI, 30.6 months-NE), 31.2 months (95% CI, 22.6 months-NE), and 26.5 months (95% CI, 20.2 months-NE), respectively. Across all subgroups, enfortumab vedotin was the most common second-line treatment after discontinuation of avelumab.

**Conclusions:**

Results from these descriptive subgroup analyses from JAVEMACS suggest that real-world outcomes with avelumab maintenance therapy are generally consistent regardless of 1L PBC regimen, number of 1L PBC cycles, or best response to 1L PBC. These findings further support the use of avelumab maintenance therapy as a standard of care for patients with la/mUC without disease progression after 1L PBC.

**Trial registration:**

NCT06412848.

**Supplementary Information:**

The online version contains supplementary material available at 10.1186/s12885-026-15781-1.

## Background

Avelumab, an anti-programmed death ligand 1 immune checkpoint inhibitor, was approved in Japan on 24 February 2021 as maintenance therapy for patients with unresectable locally advanced or metastatic urothelial carcinoma (la/mUC) without disease progression following platinum-based chemotherapy (PBC) and is recommended in clinical practice guidelines published by the Japanese Urological Association [1, 2]. Approval was based on results from the JAVELIN Bladder 100 phase III trial, in which avelumab first-line (1L) maintenance plus best supportive care (BSC) significantly prolonged overall survival (OS) and progression-free survival (PFS) vs BSC alone in patients with la/mUC that had not progressed with 1L PBC [3, 4]. Avelumab 1L maintenance also demonstrated acceptable long-term safety [3]. In post hoc analyses from the JAVELIN Bladder 100 trial, benefits with avelumab were generally consistent across subgroups defined by 1L PBC regimen (gemcitabine + cisplatin [GC] or gemcitabine + carboplatin [GCarbo]), number of cycles of 1L PBC (4-6), and best overall response to 1L PBC (complete response [CR], partial response [PR], or stable disease [SD]) [5–8]. Additionally, in a post hoc analysis in patients enrolled in Japan (*n* = 73), efficacy and safety findings were generally consistent with the overall trial population [9].

The reproducibility of results obtained in clinical trials in real-world clinical practice cannot be assumed as populations can vary. For example, patients included in clinical trials are typically younger than those in real-world clinical practice (mean difference in median age, −6.5 years) [10]. In Japan, the median age of patients with UC is > 70 years, and the proportion of patients aged ≥ 80 years is increasing annually [11]. Furthermore, aging is a potential factor that can contribute to declining renal function, leading to a decrease in the proportion of patients eligible for cisplatin. These differences may influence the 1L PBC regimens received and the duration of treatment (i.e., number of cycles of 1L PBC) in real-world clinical practice and in turn may impact outcomes with avelumab treatment.

To assess whether results from the JAVELIN Bladder 100 trial were similar in Japanese real-world clinical practice, we conducted the JAVEMACS chart review study, which included patients with la/mUC who initiated avelumab maintenance therapy in Japan between February 2021 and December 2023. Results from the primary analysis in the overall population (*N* = 350) indicated that avelumab maintenance therapy offered long-term survival benefits in real-world clinical practice; median OS from the start of avelumab was 31.8 months, and median PFS was 7.4 months [12]. Here, we report descriptive subgroup analyses from JAVEMACS, assessing outcomes with avelumab maintenance therapy in protocol-specified subgroups defined by 1L PBC regimen, number of cycles of 1L PBC, and best overall response to 1L PBC.

## Methods

### Study design and patients

The JAVEMACS (NCT06412848) study design has been described previously [12]. In brief, JAVEMACS was a multicenter, noninterventional, retrospective medical chart review of patients in Japan. The study included all patients at participating centers who met the eligibility criteria. Eligible patients were aged ≥ 18 years, had la/mUC that had not progressed (ongoing SD, PR, or CR) following 1L PBC, and had received their first dose of avelumab maintenance therapy between 24 February 2021 and December 2023. Data were collected retrospectively, and the follow-up period was defined as the period from the start of avelumab maintenance therapy until loss to follow-up, death due to any cause, or the end of the data collection period, whichever occurred first.

This study was conducted in accordance with the Declaration of Helsinki and the Ethical Guidelines for Life Science and Medical Research Involving Human Subjects (23 March 2021; partially revised 27 March 2023) issued by the Ministry of Education, Culture, Sports, Science and Technology; Ministry of Health, Labor and Welfare; and Ministry of Economy, Trade and Industry in Japan. All patients provided informed consent prior to inclusion in the study. The study protocol, amendments, and other relevant documents were approved by the independent ethics committee/institutional review board of the nonprofit organization MINS (Tokyo, Japan; Approval No. MINS-REC-240211) and hospital director of each site before the start of the study.

### Objectives

The primary objectives were to describe the baseline characteristics of patients who received avelumab maintenance therapy, characteristics of 1L PBC prior to avelumab maintenance, and subsequent treatments following avelumab maintenance. The secondary objective was to evaluate outcomes of avelumab maintenance and subsequent therapies, including OS and PFS. Here, protocol-specified subgroups defined by 1L PBC characteristics (PBC regimen [GC, GCarbo], number of PBC cycles [1–3, 4, 5/6, ≥ 7], and best overall response [CR, PR, or SD] to PBC) were analyzed descriptively. The choice of subgroups for number of PBC cycles was based on the eligibility criteria for the JAVELIN Bladder 100 phase III trial (4–6 prior cycles of chemotherapy) [3] and aimed to investigate patients who received a similar amount of chemotherapy to those in the clinical trial (i.e., 4 or 5/6 cycles) and patients who would not have been eligible for the trial (i.e., 1–3 or ≥ 7 cycles). An exploratory univariate analysis to evaluate predictive factors for OS and PFS from the start of avelumab was also performed; this was not adjusted for multiplicity. Within the univariate analysis, subgroups based on eligibility were also assessed, with eligibility determined based on characteristics at the start of 1L PBC and using published criteria. Cisplatin ineligibility was defined as meeting any of the following criteria: Eastern Cooperative Oncology Group performance status (ECOG PS) ≥ 2, creatinine clearance of < 60 mL/min (determined using the Cockcroft-Gault formula), grade ≥ 2 hearing loss, grade ≥ 2 peripheral neuropathy, and/or class III or worse New York Heart Association heart failure. Platinum ineligibility was defined as meeting any of the following criteria: ECOG PS ≥ 3, creatinine clearance of < 30 mL/min, or ECOG PS 2 and creatinine clearance of < 45 mL/min [13, 14]. These classifications were protocol-specified and applied retrospectively using baseline characteristics at the start of 1L PBC and do not imply that investigators used these criteria for treatment selection.

### Statistical analysis

This study was descriptive in nature. Continuous variables were summarized using descriptive statistics, and categorical variables were summarized by frequency counts and percentages. Time-to-event analyses were conducted using the Kaplan–Meier method, and corresponding 95% CIs were calculated using the Greenwood formula. Exploratory univariate Cox regression analyses of OS and PFS across the protocol-specified PBC-characteristic subgroups were performed to calculate hazard ratios and corresponding 95% CIs. P-values for these analyses are not reported to avoid any overinterpretation. To investigate potential heterogeneity within the RECIST-defined SD definition, we performed an exploratory, hypothesis-generating analysis by stratifying best percentage change in target lesions per investigator assessment. For this we defined a ≥ 0% to < 30% decrease in tumor size as a “shrinking SD” and a > 0% to < 20% increase in tumor size as a “growing SD”; both within the overall RECIST SD definition. This stratification was informed by prior reports in other tumors that SD is heterogeneous and that subsets of patients have differential outcomes to immunotherapy based on the depth and direction of tumor change [15, 16]. Reasons for discontinuation of avelumab maintenance (e.g., ongoing response, disease progression, adverse events/toxicity, physician or patient decision) and standardized post-discontinuation disease assessments were not prespecified in the case report form and therefore were not systematically collected across centers. For exploratory purposes the following intervals were summarized: the interval from the last dose of avelumab to the last confirmation of a patient being alive or their death in those who did not receive second-line (2L) treatment, and the interval from the last dose of avelumab to the start of 2L treatment in patients who received subsequent treatment. All analyses were performed using SAS® Software version 9.4 or later.

## Results

Between February 2021 and December 2023, 350 patients at 26 centers in Japan, including university hospitals and cancer institutes, had received ≥ 1 dose of avelumab maintenance therapy and met the study’s eligibility criteria. At data cutoff (June 2024), in the overall population, median observation time from the start of avelumab was 14.6 months (IQR, 9.7–23.9 months). Median duration of avelumab treatment was 14.3 weeks (IQR, 7.1–30.9 weeks). A total of 200 patients received any 2L treatment; in these patients, the median treatment-free interval between avelumab and 2L treatment was 1.0 months (IQR, 0.7–2.1 months). In the 133 patients treated with 2L enfortumab vedotin, the median treatment-free interval following avelumab was 0.9 months (IQR, 0.7–1.7 months). In the 40 patients who received 2L PBC (GC or GCarbo), the median treatment-free interval following avelumab was 1.0 months (0.6–2.4 months).

### Subgroups defined by 1L chemotherapy regimen

The prior 1L PBC regimen was GC in 196 patients (56.0%) and GCarbo in 116 (33.1%). Compared with the GC subgroup, the GCarbo subgroup included a higher proportion of patients who were aged ≥ 75 years (51.7% vs 36.7%), had an ECOG PS of ≥ 1 (25.0% vs 11.7%), and had upper tract primary tumors (59.5% vs 42.9%) (Table 1).

**Table 1:** Baseline characteristics at the start of avelumab maintenance therapy and characteristics of 1L PBC in subgroups defined by 1L PBC characteristics

	**1L PBC regimen**						
	**Cisplatin + gemcitabine (*n* = 196)**				**Carboplatin + gemcitabine (*n* = 116)**		
Age							
Median (range), years	72.0 (43–87)				75.0 (48–93)		
≥ 75 years, n (%)	72 (36.7)				60 (51.7)		
ECOG PS, n (%)							
0	171 (87.2)				85 (73.3)		
≥ 1	23 (11.7)				29 (25.0)		
Unknown	2 (1.0)				2 (1.7)		
Primary tumor location, n (%)							
Renal pelvis/ureter	84 (42.9)				69 (59.5)		
Bladder	110 (56.1)				46 (39.7)		
Urethra	2 (1.0)				1 (0.9)		
Radical surgery history, n (%)							
Yes	81 (41.3)				47 (40.5)		
No	115 (58.7)				69 (59.5)		
Metastatic site, n (%)^a^							
Visceral	64 (32.7)				38 (32.8)		
Liver	12 (6.1)				6 (5.2)		
Cisplatin/platinum eligibility, n (%)^b,c^							
Cisplatin eligible	78 (39.8)				5 (4.3)		
Cisplatin ineligible/platinum eligible	91 (46.4)				76 (65.5)		
Platinum ineligible	2 (1.0)				26 (22.4)		
Unknown	25 (12.8)				9 (7.8)		
Number of 1L PBC cycles, n (%)							
1–3	37 (18.9)				26 (22.4)		
4	109 (55.6)				71 (61.2)		
5/6	38 (19.4)				15 (12.9)		
≥ 7	12 (6.1)				4 (3.4)		
Platinum dose reduction, n (%)	74 (37.8)				25 (21.6)		
First cycle in which platinum dose reduction occurred, median (IQR)	1.5 (1–2)				2 (2–2)		
Best overall response to 1L PBC, n (%)							
CR	21 (10.7)				8 (6.9)		
PR	103 (52.6)				50 (43.1)		
SD	72 (36.7)				58 (50.0)		
SD (≥ 0% and < 30%)	35 (17.9)				29 (25.0)		
SD (> − 20% and < 0%)	12 (6.1)				9 (7.8)		
SD (reduction rate unclear)	25 (12.8)				20 (17.2)		
	**Number of 1L PBC cycles**						
	**1–3 cycles (*n* = 68)**	**4 cycles (*n* = 205)**		**5/6 cycles (*n* = 61)**			** ≥ 7 cycles (*n* = 16)**
Age							
Median (range), years	74.5 (50–93)	73.0 (43–87)		71.0 (47–88)			70.5 (48–87)
≥ 75 years, n (%)	34 (50.0)	85 (41.5)		22 (36.1)			3 (18.8)
ECOG PS, n (%)							
0	50 (73.5)	172 (83.9)		48 (78.7)			14 (87.5)
≥ 1	18 (26.5)	29 (14.1)		13 (21.3)			2 (12.5)
Unknown	0	4 (2.0)		0			0
Primary tumor location, n (%)							
Renal pelvis/ureter	29 (42.6)	103 (50.2)		28 (45.9)			8 (50.0)
Bladder	39 (57.4)	99 (48.3)		31 (50.8)			8 (50.0)
Urethra	0	3 (1.5)		2 (3.3)			0
Radical surgery history, n (%)							
Yes	36 (52.9)	77 (37.6)		19 (31.1)			8 (50.0)
No	32 (47.1)	128 (62.4)		42 (68.9)			8 (50.0)
Metastatic site, n (%)^a^							
Visceral	27 (39.7)	65 (31.7)		22 (36.1)			4 (25.0)
Liver	6 (8.8)	11 (5.4)		6 (9.8)			0
Cisplatin/platinum eligibility, n (%)^b,d^							
Cisplatin eligible	16 (23.5)	50 (24.4)		26 (42.6)			8 (50.0)
Cisplatin ineligible/platinum eligible	41 (60.3)	116 (56.6)		24 (39.3)			4 (25.0)
Platinum ineligible	5 (7.4)	19 (9.3)		5 (8.2)			1 (6.3)
Unknown	6 (8.8)	20 (9.8)		6 (9.8)			3 (18.8)
1L PBC regimen, n (%)							
Cisplatin + gemcitabine	37 (54.4)	109 (53.2)		38 (62.3)			12 (75.0)
Carboplatin + gemcitabine	26 (38.2)	71 (34.6)		15 (24.6)			4 (25.0)
ddMVAC	4 (5.9)	22 (10.7)		6 (9.8)			0
Other	1 (1.5)	3 (1.5)		2 (3.3)			0
Platinum dose reduction, n (%)	26 (38.2)	62 (30.2)		22 (36.1)			4 (25.0)
First cycle in which platinum dose reduction occurred, median (IQR)	1 (1–2)	2 (1–2)		2 (1–3)			3 (2–5)
Best overall response to 1L PBC, n (%)							
CR	6 (8.8)	14 (6.8)		9 (14.8)			3 (18.8)
PR	27 (39.7)	107 (52.2)		35 (57.4)			11 (68.8)
SD	35 (51.5)	84 (41.0)		17 (27.9)			2 (12.5)
SD (≥ 0% and < 30%)	19 (27.9)	45 (22.0)		6 (9.8)			1 (6.3)
SD (> − 20% and < 0%)	6 (8.8)	10 (4.9)		5 (8.2)			0
SD (reduction rate unclear)	10 (14.7)	29 (14.1)		6 (9.8)			1 (6.3)
	**Best overall response to 1L PBC**						
	**CR (*n* = 32)**		**PR (*n* = 180)**			**SD (*n* = 138)**	
Age							
Median (range), years	74.5 (43–87)		73.0 (47–93)			73.5 (48–89)	
≥ 75 years, n (%)	16 (50.0)		70 (38.9)			58 (42.0)	
ECOG PS, n (%)							
0	25 (78.1)		149 (82.8)			110 (79.7)	
≥ 1	7 (21.9)		30 (16.7)			25 (18.1)	
Unknown	0		1 (0.6)			3 (2.2)	
Primary tumor location, n (%)							
Renal pelvis/ureter	15 (46.9)		93 (51.7)			60 (43.5)	
Bladder	17 (53.1)		84 (46.7)			76 (55.1)	
Urethra	0		3 (1.7)			2 (1.4)	
Radical surgery history, n (%)							
Yes	22 (68.8)		65 (36.1)			53 (38.4)	
No	10 (31.3)		115 (63.9)			85 (61.6)	
Metastatic site, n (%)^a^							
Visceral	13 (40.6)		61 (33.9)			44 (31.9)	
Liver	3 (9.4)		15 (8.3)			5 (3.6)	
Cisplatin/platinum eligibility, n (%)^b,e^							
Cisplatin eligible	9 (28.1)		53 (29.4)			38 (27.5)	
Cisplatin ineligible/platinum eligible	12 (37.5)		96 (53.3)			77 (55.8)	
Platinum ineligible	5 (15.6)		14 (7.8)			11 (8.0)	
Unknown	6 (18.8)		17 (9.4)			12 (8.7)	
1L PBC regimen, n (%)							
Cisplatin + gemcitabine	21 (65.6)		103 (57.2)			72 (52.2)	
Carboplatin + gemcitabine	8 (25.0)		50 (27.8)			58 (42.0)	
ddMVAC	3 (9.4)		21 (11.7)			8 (5.8)	
Other	0		6 (3.3)			0	
Number of 1L PBC cycles, n (%)							
1–3	6 (18.8)		27 (15.0)			35 (25.4)	
4	14 (43.8)		107 (59.4)			84 (60.9)	
5/6	9 (28.1)		35 (19.4)			17 (12.3)	
≥ 7	3 (9.4)		11 (6.1)			2 (1.4)	
Platinum dose reduction, n (%)	8 (25.0)		62 (34.4)			44 (31.9)	
First cycle in which platinum dose reduction occurred, median (IQR)	2 (2–4)		1 (1–2)			2 (1–3)	

*1L* first line, *CR* complete response, *ddMVAC* dose-dense methotrexate, vinblastine, doxorubicin, and cisplatin, *ECOG PS* Eastern Cooperative Oncology Group performance status, *NYHA* New York Heart Association, *PBC* platinum-based chemotherapy, *PR* partial response, *SD* stable disease

^a^Metastatic site determined at the start of 1L PBC

^b^Eligibility determined based on characteristics at the start of 1L PBC. Cisplatin ineligibility was defined as meeting any of the following criteria: ECOG PS ≥ 2, creatinine clearance of < 60 mL/min, grade ≥ 2 hearing loss, grade ≥ 2 peripheral neuropathy, and/or class III or worse NYHA heart failure. Platinum ineligibility was defined as meeting any of the following criteria: ECOG PS ≥ 3, creatinine clearance of < 30 mL/min, or ECOG PS 2 and creatinine clearance of < 45 mL/min [13, 14]

^c^In the cisplatin + gemcitabine and carboplatin + gemcitabine subgroups, respectively, cisplatin ineligibility was due to creatinine clearance < 60 to ≥ 30 mL/min in 88 (96.7%) and 76 patients (100%), ECOG PS 2 in 5 (5.5%) and 0, and grade ≥ 2 hearing impairment in 2 (2.2%) and 2 (2.6%). No patient had grade ≥ 2 peripheral neuropathy or NYHA class ≥ III heart failure at the start of 1L PBC. Some patients had > 1 reason. Platinum ineligibility was due to creatinine clearance < 30 mL/min in 2 (100%) and 25 (96.2%) and ECOG PS 3 in 0 and 1 (3.8%). No patient had creatinine clearance of < 45 to ≥ 30 mL/min and ECOG PS 2 at the start of 1L PBC

^d^In the 1–3, 4, 5/6, and ≥ 7 cycles subgroups, respectively, cisplatin ineligibility was due to creatinine clearance < 60 to ≥ 30 mL/min in 41 (100%), 113 (97.4%), 23 (95.8%), and 4 patients (100%), ECOG PS 2 in 1 (2.4%), 4 (3.4%), 0, and 0, and grade ≥ 2 hearing impairment in 2 (4.9%), 2 (1.7%), 1 (4.2%), and 0. No patient had grade ≥ 2 peripheral neuropathy or NYHA class ≥ III heart failure at the start of 1L PBC. Some patients had > 1 reason. Platinum ineligibility was due to creatinine clearance < 30 mL/min in 5 (100%), 17 (89.5%), 5 (100%), and 1 (100%), and ECOG PS 3 in 0, 2 (10.5%), 0, and 0. No patient had creatinine clearance of < 45 to ≥ 30 mL/min and ECOG PS 2 at the start of 1L PBC

^e^In the CR, PR, and SD subgroups, respectively, cisplatin ineligibility was due to creatinine clearance < 60 to ≥ 30 mL/min in 12 (100%), 93 (96.9%), and 76 patients (98.7%), ECOG PS 2 in 0, 2 (14.3%), and 0, and grade ≥ 2 hearing impairment in 0, 5 (5.2%), and 1 (1.3%). No patient had grade ≥ 2 peripheral neuropathy or NYHA class ≥ III heart failure at the start of 1L PBC. Some patients had > 1 reason. Platinum ineligibility was due to creatinine clearance < 30 mL/min in 5 (100%), 12 (85.7%), and 11 (100%), and ECOG PS 3 in 0, 2 (14.3%), and 0. No patient had creatinine clearance of < 45 to ≥ 30 mL/min and ECOG PS 2 at the start of 1L PBC

At data cutoff, 34 patients (17.3%) in the GC subgroup and 23 patients (19.8%) in the GCarbo subgroup were still receiving avelumab maintenance therapy; 41 (20.9%) and 30 patients (25.9%), respectively, had discontinued avelumab without subsequent treatment (Table 2). In the GC subgroup, among patients who discontinued avelumab without receiving subsequent treatment, 23 of 41 patients were alive, 14 had died, and 4 were lost to follow-up at data cutoff. The median duration of avelumab treatment in these 41 patients was 3.4 months (IQR, 1.0–6.9 months) and the median time from avelumab discontinuation to the last confirmation of being alive or death was 5.1 months (IQR, 2.9–11.5 months). In the GCarbo subgroup, 12 of 30 patients who discontinued avelumab without receiving subsequent treatment were alive, 15 had died, and 3 were lost to follow-up at data cutoff. The median duration of avelumab treatment was 3.6 months (IQR, 1.4–6.8 months), and the median time from discontinuation to the last confirmation of being alive or death was 3.4 months (IQR, 1.3–7.7 months). In patients who discontinued avelumab treatment, 121 (74.7%) in the GC subgroup and 63 (67.7%) in the GCarbo subgroup had received 2L treatment. The most common 2L treatment in both subgroups was enfortumab vedotin (GC, *n* = 78 [64.5%]; GCarbo, *n* = 44 [69.8%]).

**Table 2:** Patient disposition and subsequent treatments after avelumab maintenance in subgroups defined by 1L PBC characteristics

	**1L PBC regimen**					
	**Cisplatin + gemcitabine (*n* = 196)**			**Carboplatin + gemcitabine (*n* = 116)**		
Ongoing avelumab maintenance therapy, n (%)	34 (17.3)			23 (19.8)		
Discontinued avelumab maintenance and received no 2L treatment, n (%)	41 (20.9)			30 (25.9)		
Discontinued avelumab maintenance and received 2L treatment, n (%)^a^	121 (74.7)			63 (67.7)		
2L treatment regimen, n (%)^b^						
EV	78 (64.5)			44 (69.8)		
Cisplatin + gemcitabine	24 (19.8)			0		
Carboplatin + gemcitabine	2 (1.7)			13 (20.6)		
ddMVAC	0			0		
Pembrolizumab	10 (8.3)			4 (6.3)		
Others	7 (5.8)			2 (3.2)		
	**Number of 1L PBC cycles**					
	**1–3 cycles (*n* = 68)**	**4 cycles (*n* = 205)**		**5/6 cycles (*n* = 61)**		** ≥ 7 cycles (*n* = 16)**
Ongoing avelumab maintenance therapy, n (%)	5 (7.4)	44 (21.5)		16 (26.2)		2 (12.5)
Discontinued avelumab maintenance and received no 2L treatment, n (%)	22 (32.4)	46 (22.4)		11 (18.0)		4 (25.0)
Discontinued avelumab maintenance and received 2L treatment, n (%)^a^	41 (65.1)	115 (71.4)		34 (75.6)		10 (71.4)
2L treatment regimen, n (%)^b^						
EV	29 (70.7)	78 (67.8)		20 (58.8)		6 (60.0)
Cisplatin + gemcitabine	4 (9.8)	12 (10.4)		9 (26.5)		0
Carboplatin + gemcitabine	4 (9.8)	9 (7.8)		2 (5.9)		0
ddMVAC	0	1 (0.9)		0		0
Pembrolizumab	3 (7.3)	11 (9.6)		1 (2.9)		2 (20.0)
Others	1 (2.4)	4 (3.5)		2 (5.9)		2 (20.0)
	**Best overall response to 1L PBC**					
	**CR (*n* = 32)**		**PR (*n* = 180)**		**SD (*n* = 138)**	
Ongoing avelumab maintenance therapy, n (%)	13 (40.6)		33 (18.3)		21 (15.2)	
Discontinued avelumab maintenance and received no 2L treatment, n (%)	2 (6.3)		46 (25.6)		35 (25.4)	
Discontinued avelumab maintenance and received 2L treatment, n (%)^a^	17 (89.5)		101 (68.7)		82 (70.1)	
2L treatment regimen, n (%)^b^						
EV	11 (64.7)		64 (63.4)		58 (70.7)	
Cisplatin + gemcitabine	3 (17.6)		17 (16.8)		5 (6.1)	
Carboplatin + gemcitabine	1 (5.9)		9 (8.9)		5 (6.1)	
ddMVAC	0		1 (1.0)		0	
Pembrolizumab	1 (5.9)		5 (5.0)		11 (13.4)	
Others	1 (5.9)		5 (5.0)		3 (3.7)	

*1L* first line, *2L* second line, *CR* complete response, *ddMVAC* dose-dense methotrexate, vinblastine, doxorubicin, and cisplatin, *EV* enfortumab vedotin, *PBC* platinum-based chemotherapy, *PR* partial response, *SD* stable disease

^a^Percentage of patients who discontinued avelumab maintenance therapy

^b^Percentage of patients receiving 2L treatment

**Fig. 1:**
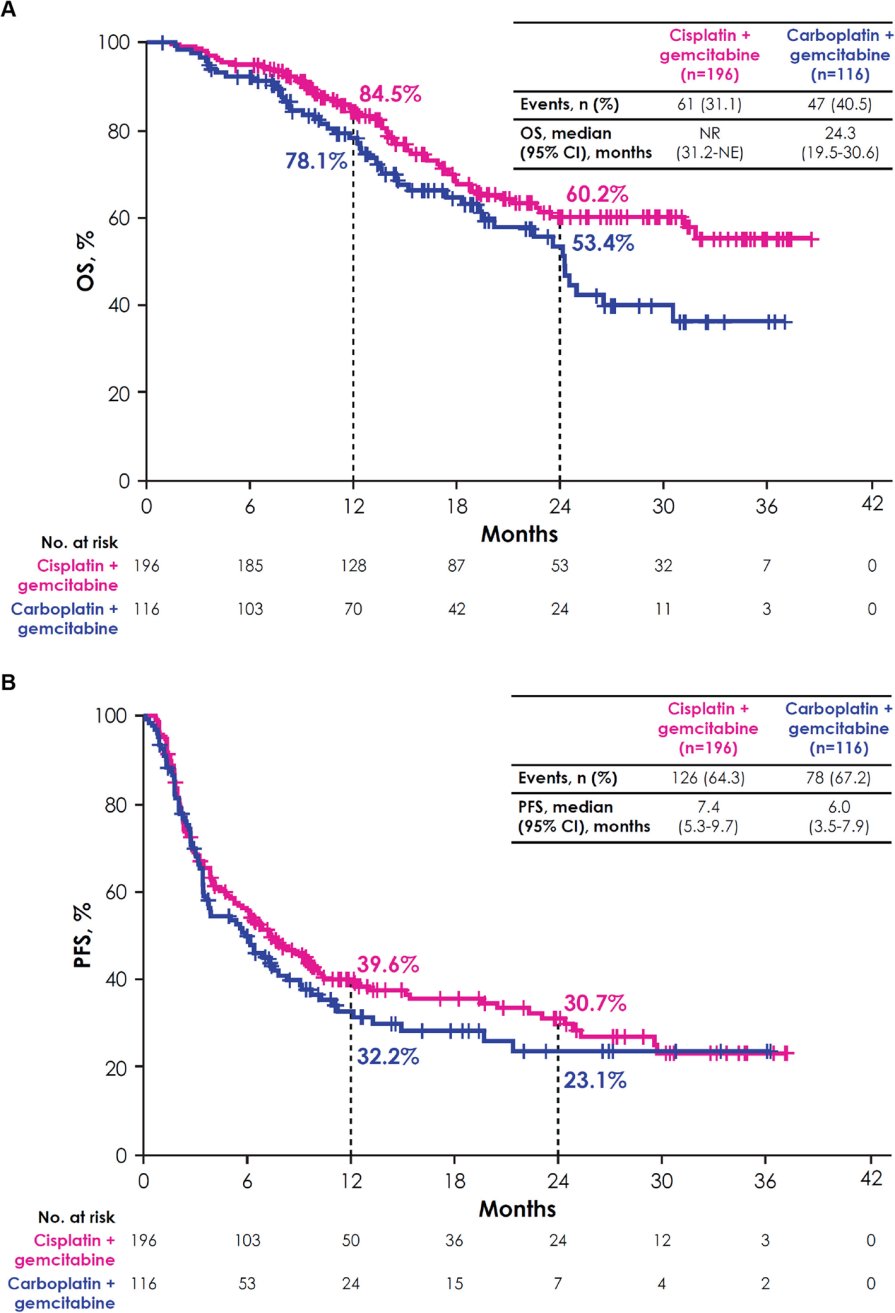
OS (**A**) and PFS (**B**) from start of avelumab maintenance by 1L PBC regimen. *1L* first line, *NE* not estimable, *NR* not reached, *OS* overall survival, *PBC* platinum-based chemotherapy, *PFS* progression-free survival

**Fig. 2:**
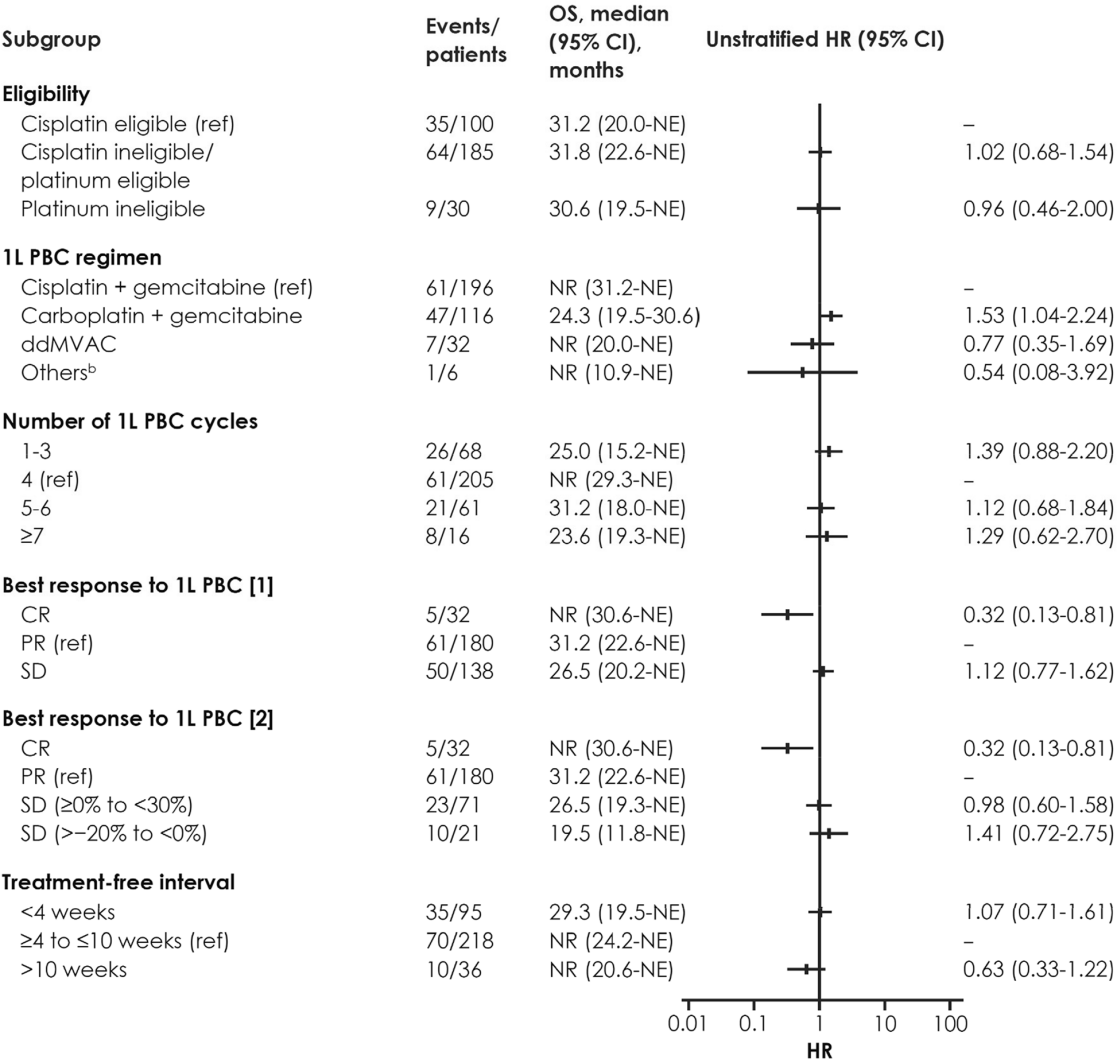
Univariate analysis of 1L PBC factors associated with OS from start of avelumab maintenance. *1L* first line, *CR* complete response, *ddMVAC* dose-dense methotrexate, vinblastine, doxorubicin, and cisplatin, *HR* hazard ratio, *NE* not estimable, *NR* not reached, *OS* overall survival, *PBC* platinum-based chemotherapy, *PR* partial response, *ref* reference, *SD* stable disease. ^a^Univariate analyses are exploratory and were not adjusted for multiplicity ^b^Other 1L PBC regimens included nedaplatin + gemcitabine (*n* = 4), cisplatin + etoposide (*n* = 1), and carboplatin + etoposide (*n* = 1)

Median OS was not reached (95% CI, 31.2 months-not estimable [NE]) in the GC subgroup and 24.3 months (95% CI, 19.5–30.6 months) in the GCarbo subgroup; 2-year OS rates were 60.2% and 53.4%, respectively (Fig. 1A). In the GC and GCarbo subgroups, respectively, median PFS was 7.4 months (95% CI, 5.3–9.7 months) and 6.0 months (95% CI, 3.5–7.9 months) (Fig. 1B). In univariate analyses, 1L PBC regimen did not appear to be associated with OS (Fig. 2) or PFS (Supplementary Fig. 1).

### Subgroups defined by number of 1L PBC cycles

The number of 1L PBC cycles was 1–3 in 68 patients (19.4%), 4 in 205 (58.6%), 5/6 in 61 (17.4%), and ≥ 7 in 16 (4.6%). Subgroups that received fewer cycles of 1L PBC had a higher proportion of patients aged ≥ 75 years and a lower proportion of patients who received GC and had CR to 1L PBC (Table 1).

At data cutoff, 5 patients (7.4%) in the 1–3 cycles subgroup, 44 (21.5%) in the 4 cycles subgroup, 16 (26.2%) in the 5/6 cycles subgroup, and 2 (12.5%) in the ≥ 7 cycles subgroup were still receiving avelumab maintenance therapy; 22 (32.4%), 46 (22.4%), 11 (18.0%), and 4 patients (25.0%), respectively, had discontinued avelumab without subsequent treatment (Table 2). In patients who discontinued avelumab treatment, 41 (65.1%) in the 1–3 cycles subgroup, 115 (71.4%) in the 4 cycles subgroup, 34 (75.6%) in the 5/6 cycles subgroup, and 10 (71.4%) in the ≥ 7 cycles subgroup had received 2L treatment. The most common 2L treatment in all subgroups defined by number of 1L PBC cycles was enfortumab vedotin (1–3 cycles, *n* = 29 [70.7%]; 4 cycles, *n* = 78 [67.8%]; 5/6 cycles, *n* = 20 [58.8%]; ≥ 7 cycles, *n* = 6 [60.0%]).

**Fig. 3:**
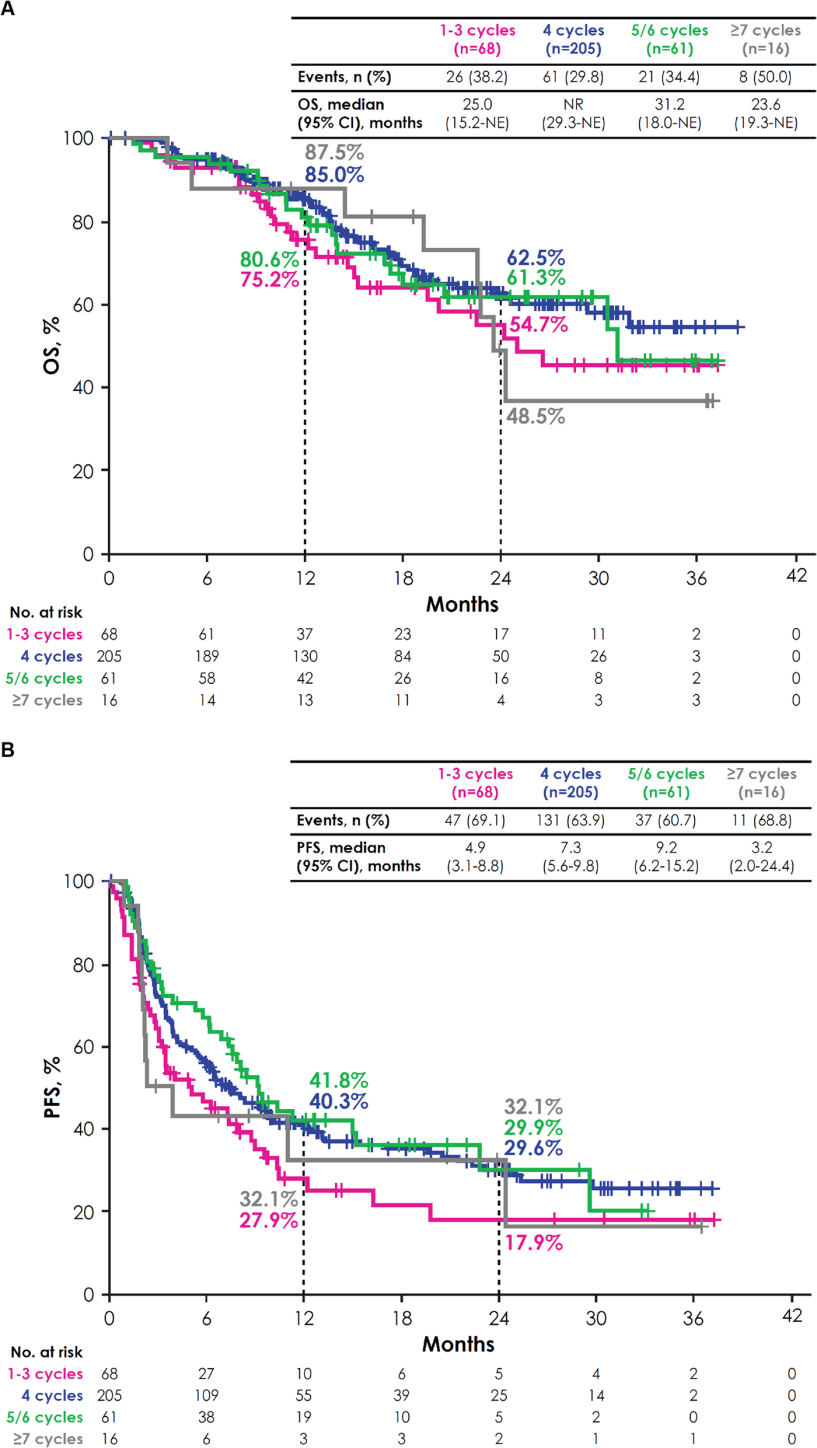
OS (**A**) and PFS (**B**) from start of avelumab by number of 1L PBC cycles. *1L* first line, *NE* not estimable, *NR* not reached, *OS* overall survival, *PBC* platinum-based chemotherapy, *PFS* progression-free survival

Median OS was 25.0 months (95% CI, 15.2 months-NE) in the 1–3 cycles subgroup, not reached (95% CI, 29.3 months-NE) in the 4 cycles subgroup, 31.2 months (95% CI, 18.0 months-NE) in the 5/6 cycles subgroup, and 23.6 months (95% CI, 19.3 months-NE) in the ≥ 7 cycles subgroup; 2-year OS rates were 54.7%, 62.5%, 61.3%, and 48.5%, respectively (Fig. 3A). In the 1–3, 4, 5/6, and ≥ 7 cycles subgroups, respectively, median PFS was 4.9 months (95% CI, 3.1–8.8 months), 7.3 months (95% CI, 5.6–9.8 months), 9.2 months (95% CI, 6.2–15.2 months), and 3.2 months (95% CI, 2.0–24.4 months) (Fig. 3B). In univariate analyses, the number of 1L PBC cycles did not appear to be associated with OS (Fig. 2) or PFS (Supplementary Fig. 1).

### Subgroups defined by best overall response to 1L PBC

Best overall response to 1L PBC was CR in 32 patients (9.1%), PR in 180 (51.4%), and SD in 138 (39.4%). A higher proportion of patients were aged ≥ 75 years in the CR subgroup (50.0%) than in the PR and SD subgroups (38.9% and 42.0%, respectively) (Table 1). Additionally, a higher proportion of patients had received 1L GC in the CR subgroup (65.6%) vs the PR and SD subgroups (57.2% and 52.2%, respectively).

At data cutoff, 13 patients (40.6%) in the CR subgroup, 33 (18.3%) in the PR subgroup, and 21 (15.2%) in the SD subgroup were still receiving avelumab maintenance therapy; 2 (6.3%), 46 (25.6%), and 35 patients (25.4%), respectively, had discontinued avelumab without subsequent treatment (Table 2). Among patients who discontinued avelumab without receiving subsequent systemic treatment, in the CR subgroup, 1 of 2 patients were alive, and 1 had died at data cutoff. For these 2 patients, the duration of avelumab treatment was 2.7 and 14.9 months, respectively, and the time from avelumab discontinuation to the last confirmation of being alive or death was 30.1 and 15.6 months. In the PR subgroup, 25 of 46 patients who discontinued avelumab without receiving subsequent systemic treatment were alive, 17 had died, and 4 were lost to follow-up at data cutoff. The median duration of avelumab treatment in these 46 patients was 4.6 months (IQR, 1.5–8.1 months), and the median time from avelumab discontinuation to the last confirmation of being alive or death was 4.1 months (IQR, 2.0–14.2 months). In the SD subgroup, 15 of 35 patients who discontinued avelumab without receiving subsequent systemic treatment were alive, 13 had died, and 7 were lost to follow-up at data cutoff. The median duration of avelumab treatment in these 35 patients was 3.4 months (IQR, 0.7–6.2 months), and the median time from discontinuation to the last confirmation of being alive or death was 3.9 months (IQR, 1.8–7.5 months). In patients who discontinued avelumab treatment, 17 (89.5%) in the CR subgroup, 101 (68.7%) in the PR subgroup, and 82 (70.1%) in the SD subgroup had received 2L treatment. The most common 2L treatment in all subgroups defined by best overall response to 1L PBC was enfortumab vedotin (CR, *n* = 11 [64.7%]; PR, *n* = 64 [63.4%]; SD, *n* = 58 [70.7%]).

**Fig. 4:**
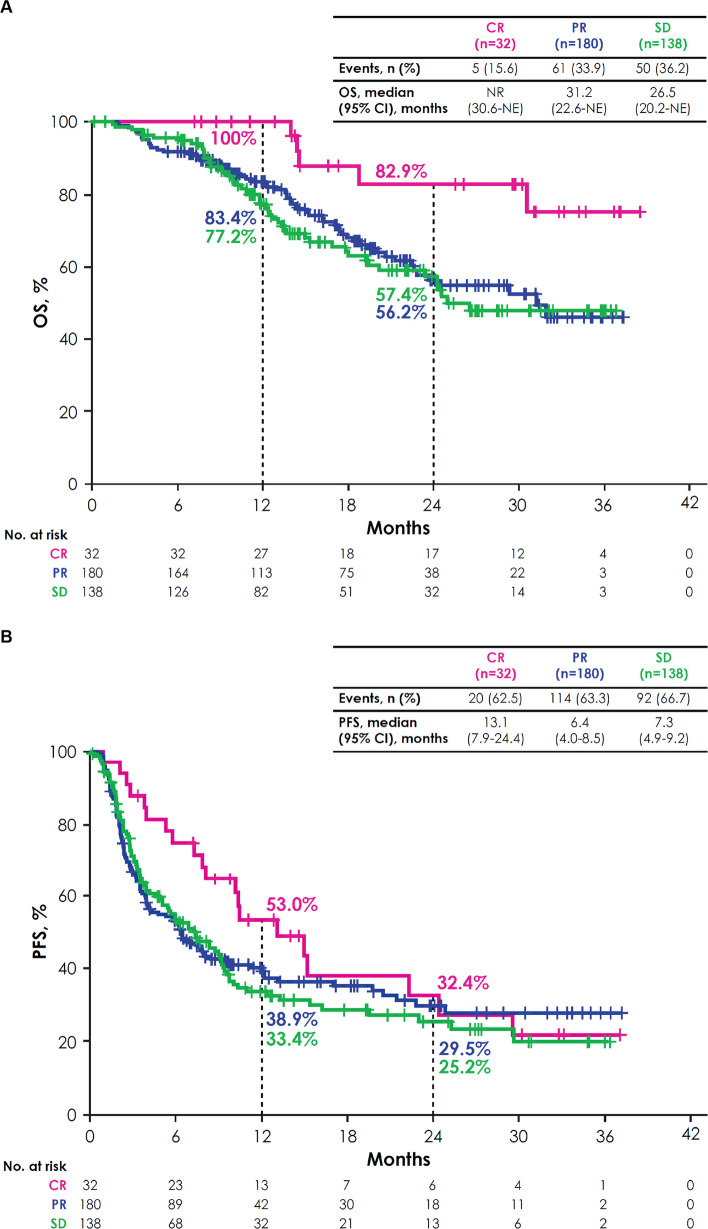
OS (**A**) and PFS (**B**) from start of avelumab by best response to 1L PBC. *1L* first line, *CR* complete response, *NE* not estimable, *NR* not reached, *OS* overall survival, *PBC* platinum-based chemotherapy, *PFS* progression-free survival, *PR* partial response, *SD* stable disease

Median OS was not reached (95% CI, 30.6 months-NE) in the CR subgroup, 31.2 months (95% CI, 22.6 months-NE) in the PR subgroup, and 26.5 months (95% CI, 20.2 months-NE) in the SD subgroup; 2-year OS rates were 82.9%, 56.2%, and 57.4%, respectively (Fig. 4A). In a univariate analysis, best overall response to 1L PBC appeared to be associated with OS (Fig. 2). In the CR, PR, and SD subgroups, respectively, median PFS was 13.1 months (95% CI, 7.9–24.4 months), 6.4 months (95% CI, 4.0–8.5 months), and 7.3 months (95% CI, 4.9–9.2 months) (Fig. 4B). In a univariate analysis, best overall response to 1L PBC did not appear to be associated with PFS (Supplementary Fig. 1). In the subgroup of patients with SD with 1L PBC, median OS was 26.5 months (95% CI, 19.3 months-NE) in patients with ≥ 0% and < 30% decrease in tumor size (shrinking SD; *n* = 71) and 19.5 months (95% CI, 11.8 months-NE) in patients with > 0% and < 20% increase in tumor size (growing SD; *n* = 21) (Supplementary Fig. 2A); median PFS was 6.9 months (95% CI, 4.2–9.4 months) and 2.8 months (95% CI, 1.9–5.1 months), respectively (Supplementary Fig. 2B).

## Discussion

Previous results from JAVEMACS provided insights into patient characteristics, treatment patterns, and outcomes in a large population of patients with la/mUC who received avelumab maintenance therapy in a real-world setting [12]. The descriptive protocol-specified subgroup analyses presented here indicate that real-world outcomes with avelumab maintenance therapy are generally consistent regardless of 1L PBC regimen, number of 1L PBC cycles, or best overall response to 1L PBC.

Overall, the majority of patients had received 1L GC (56.0%), 4 cycles of PBC (58.6%), and had a PR to PBC (51.4%). Patient characteristics were generally similar across the various PBC subgroups. However, fewer patients who received GC vs GCarbo were aged ≥ 75 years (36.7% vs 51.7%, respectively) or had an Eastern Cooperative Oncology Group performance status of ≥ 1 (11.7% vs 25.0%). Of note, almost half of patients who had received GC (47.4%) were deemed ineligible for cisplatin and/or platinum prior to receiving PBC, per published criteria [13, 14]. In real-world clinical practice, treatment decisions are often shaped by multiple factors such as physician judgment, patient preferences, and healthcare accessibility, rather than being determined solely according to published eligibility criteria. This is reflected in a recent phase III trial that classified patients with glomerular filtration rate ≥ 50 mL/min, instead of creatinine clearance ≥ 60 mL/min per published criteria, as cisplatin eligible based on the investigator’s clinical judgment [13, 17]. Split-dose cisplatin is an additional treatment option for patients with comorbidities, poor performance status, and a range of creatinine clearance levels [18], increasing the number of patients who receive cisplatin in clinical practice. In JAVEMACS, 37.8% of patients who had received 1L GC had their platinum dose reduced, most commonly in cycle 1 or 2. The proportion of patients who had received 1L GC was higher in the subgroup that had CR with 1L PBC (65.6%) than in the subgroups that had PR (57.2%) or SD (52.2%).

Outcomes with avelumab in the GC subgroup were generally consistent with those in the overall population (median OS: not reached [95% CI, 31.2 months-NE] vs 31.8 months) [12]. Compared with the GC subgroup, the GCarbo subgroup had a numerically lower median OS (24.3 months); however, in an exploratory univariate analysis, 1L PBC regimen was not significantly associated with OS or PFS. These results are similar to those seen in the avelumab + BSC arm of the JAVELIN Bladder 100 trial, in which patients who received 1L GC had a numerically higher median OS than those who received GCarbo (25.1 vs 20.8 months, respectively) [7]. Patients who are eligible for cisplatin-based chemotherapy tend to have a better prognosis than those who are ineligible [19, 20], which may have influenced the improved outcomes observed with GC vs GCarbo. The efficacy of GCarbo in cisplatin-ineligible patients has been reported previously, with a median OS of 9.3 months and a disease control rate of 73.9% in a phase II/III trial [21], indicating that a large proportion of cisplatin-ineligible patients could obtain a potential survival benefit with subsequent avelumab maintenance therapy.

The optimal number of 1L PBC cycles is unknown. In a retrospective study assessing outcomes with 1L PBC by number of cycles, OS was not significantly different in patients who received 4 vs 6 cycles of 1L PBC [22]. Similarly, in exploratory analyses of JAVELIN Bladder 100, outcomes with avelumab maintenance therapy in subgroups that received 4 or 6 cycles of 1L PBC were generally consistent with those in the overall trial population [6]. In JAVEMACS, some patients (24.0%) had received < 4 and > 6 cycles of 1L PBC, but the majority had received 4 cycles (58.6%). Outcomes with avelumab maintenance were generally consistent across 1L PBC cycle subgroups, although median OS was numerically higher in patients who had received 4 and 5/6 cycles (not reached and 31.2 months, respectively) compared with patients who received 1–3 and ≥ 7 cycles (25.0 and 23.6 months, respectively). However, these results should be interpreted with caution because patient numbers in some of the subgroups were relatively small and our analyses did not account for other potential confounding factors between these subgroups. Nevertheless, our analyses provide insights into avelumab 1L maintenance treatment following chemotherapy durations beyond those reported in JAVELIN Bladder 100 (i.e., < 4 and > 6 cycles). While the optimal number of 1L PBC cycles is unknown, studies suggest a possible benefit of transitioning to avelumab maintenance therapy after 4 cycles due to limited additional efficacy with more cycles and to reduce the risk of cumulative toxicity [22–24]. Additionally, the randomized phase II trial, DISCUS, recently reported that 3 cycles of 1L PBC prior to avelumab maintenance therapy was associated with significantly better quality of life (primary endpoint) than 6 cycles, with no significant difference in efficacy; the trial is ongoing and a final OS analysis is planned [25, 26].

In JAVEMACS, patients who had CR as best overall response to 1L PBC had improved OS compared with patients who had PR or SD (median, not reached vs 31.2 and 26.5 months, respectively). These results are similar to those in the avelumab + BSC arm of the JAVELIN Bladder 100 trial, in which patients who had CR as best response to 1L PBC had a numerically higher median OS than those who had PR or SD (39.8 vs 19.2 and 22.3 months, respectively) [8]. In JAVEMACS, patients with growing SD (> 0% and < 20% increase in tumor size) had a numerically shorter OS than patients with shrinking SD (≥ 0% and < 30% decrease in tumor size; 19.5 vs 26.5 months, respectively). Although this difference was not statistically significant, it adds to current evidence that tumor response classified as SD is heterogeneous and could potentially be prognostically relevant [15, 16]. Variability in the timing of response assessments may influence categorization of response, and some patients with growing SD may have experienced prior tumor shrinkage that would have been detected if assessments were performed earlier or more frequently, thus implying that earlier initiation of avelumab maintenance could potentially improve outcomes in some patients. Additionally, in patients with growing SD, tumor response after switching to avelumab maintenance therapy should be monitored closely to ensure optimal timing of transition to the next line of therapy. To our knowledge, such stratification of SD based on tumor shrinkage or growth has not been evaluated in previous studies of avelumab maintenance therapy, including JAVELIN Bladder 100. The RECIST criteria do not define subcategories within SD; therefore, our shrinking-versus-growing SD split is exploratory and hypothesis generating. The stratification was chosen based on prior reports that patients who received immunotherapy commonly have SD and this is heterogeneous, with subsets of patients further defined by depth/direction of tumor change having varied outcomes [15, 16]. Accordingly, the stratification used in this analysis was intended to investigate whether similar within-SD directionality may be informative in predicting benefit to real-world avelumab maintenance treatment. These exploratory findings suggest further research is warranted to refine risk assessment and optimize the timing of sequential therapy in patients with SD following 1L PBC.

An important practical question is whether avelumab 1L maintenance treatment can be safely discontinued or intermittently paused, particularly in patients who achieve CR or deep PR. In prescribing information, avelumab maintenance is recommended to be continued until disease progression or unacceptable toxicity. In JAVEMACS, real-world practice in Japan seems to align with this, with patients who achieved a prior response to 1L PBC and potentially have ongoing CR or deep PR continuing with avelumab maintenance treatment. At data cutoff, 40.6% of patients in the CR subgroup, 18.3% in the PR subgroup, and 15.2% in the SD subgroup were still receiving avelumab. In patients who did not receive 2L treatment, avelumab was discontinued after a median treatment duration of approximately 3–5 months, and the subsequent median treatment-free interval was approximately 3–5 months; however, individual patterns were heterogeneous, with some patients stopping early and others experiencing more prolonged treatment or treatment-free periods. Notably, the 2 patients with CR who discontinued avelumab without 2L treatment had prolonged combined durations of avelumab treatment and subsequent treatment-free intervals (approaching approximately 30 months in total for each patient). Moreover, only 2 of 15 patients with CR to 1L PBC who did not receive 2L treatment had discontinued avelumab by data cutoff. Taken together, these observations suggest that elective early discontinuation of avelumab was uncommon in our cohort and that there was no single characteristic pattern of treatment duration or treatment-free interval that would allow us to define a shortened fixed-duration maintenance strategy. However, reasons for discontinuation of avelumab maintenance and standardized post-discontinuation disease assessments were not systematically collected across centers, and JAVEMACS was not designed to investigate the optimal duration of avelumab maintenance therapy. Further prospective studies are needed to evaluate if avelumab maintenance can be given for a fixed-duration or de-escalated in certain patients.

In patients who discontinued avelumab maintenance therapy, approximately 70% received 2L treatment, regardless of 1L PBC regimen, number of 1L PBC cycles, or best response to 1L PBC. Compared with other subgroups, a higher proportion of patients who had CR to 1L PBC received 2L treatment after discontinuation of avelumab (89.5%). In all subgroups, the most common 2L treatment received was enfortumab vedotin. The effectiveness of 2L enfortumab vedotin monotherapy in this population is supported by studies in real-world US populations in which the median OS from start of 2L enfortumab vedotin in patients who had received 1L PBC without progression and avelumab maintenance was 11.2–13.3 months [27–29], and the EV-301 phase III trial and Japanese real-world studies in patients with progression after PBC and immune checkpoint inhibitor treatment, which each reported a median OS of 12.9 months [30–32]. Exploratory analyses of treatment-free intervals in patients who discontinued avelumab and received 2L PBC indicate that, in this real-world cohort, most patients transitioned to 2L PBC after only a short interval without systemic treatment.

This study had some limitations. As data collection was retrospective, it relied on medical records, which may be missing data. The timing of evaluations and assessments, treatment selection, and timing of transition to avelumab maintenance therapy varied among patients. Evaluation of disease response was per physician assessment and may have differed at each site. Additionally, because the study was conducted in university hospitals and cancer institutes, which are likely to have a higher level of expertise in the treatment of la/mUC, these results may vary from those in general clinical practice. This study involved exploratory subgroup analyses that lacked sufficient statistical power, and univariate analyses were not adjusted for multiplicity; therefore, results should be interpreted with caution. In addition, reasons for avelumab discontinuation and post-discontinuation disease assessments were not systematically documented across centers. Consequently, the rates of discontinuation due to ongoing response vs toxicity or clinical deterioration could not be determined, and survival outcomes following avelumab discontinuation could not be assessed. The exploratory analyses of post-discontinuation intervals between avelumab and either confirmation of being alive or death or 2L treatment should therefore be interpreted with caution.

## Conclusion

Results from these protocol-defined subgroup analyses further support the use of avelumab maintenance therapy as a standard of care for patients with la/mUC without disease progression after 1L PBC, irrespective of the characteristics of, and best response to, 1L PBC. These findings suggest that PBC regimen and number of cycles can be tailored according to individual patient considerations (e.g., resolution of chemotherapy-related toxicities, treatment breaks, scheduling logistics, and healthcare professional/patient preferences) in real-world clinical practice.

## Supplementary Information

Supplementary Material 1.

## Data Availability

Any requests for data by qualified scientific and medical researchers for legitimate research purposes will be subject to Merck’s Data Sharing Policy. All requests should be submitted in writing to Merck’s data sharing portal (https://www.merckgroup.com/en/research/our-approach-to-research-and-development/healthcare/clinical-trials/commitment-responsible-data-sharing.html). When Merck has a co-research, co-development, or co-marketing or co-promotion agreement, or when the product has been out-licensed, the responsibility for disclosure might be dependent on the agreement between parties. Under these circumstances, Merck will endeavor to gain agreement to share data in response to requests.

## Abbreviations

1L: First line
2L: Second line
BSC: Best supportive care
CR: Complete response
ECOG PS: Eastern Cooperative Oncology Group performance status
GC: Gemcitabine + cisplatin
GCarbo: Gemcitabine + carboplatin
la/m: Locally advanced or metastatic
NE: Not estimable
NR: Not reached
OS: Overall survival
PBC: Platinum-based chemotherapy
PFS: Progression-free survival
PR: Partial response
SD: Stable disease
UC: Urothelial carcinoma
