# Supplementary material for: Avelumab maintenance therapy for advanced urothelial carcinoma: subgroup analyses by characteristics of first-line platinum-based chemotherapy from a real-world chart review study in Japan (JAVEMACS)
Source: BMC Cancer. 2026 Mar 26;26:575. doi: 10.1186/s12885-026-15781-1 (PMC13151371; doi:10.1186/s12885-026-15781-1)
Supplement: Supplementary file 1 — Supplementary Material 1. [file 12885_2026_15781_MOESM1_ESM.docx]

**Supplement to *Avelumab maintenance therapy for advanced urothelial carcinoma: subgroup analyses by characteristics of first-line platinum-based chemotherapy from a real-world chart review study in Japan (JAVEMACS)***

**Supplementary Figure 1. Univariate analysis of 1L PBC factors associated with PFS from start of avelumab maintenance.^a^**


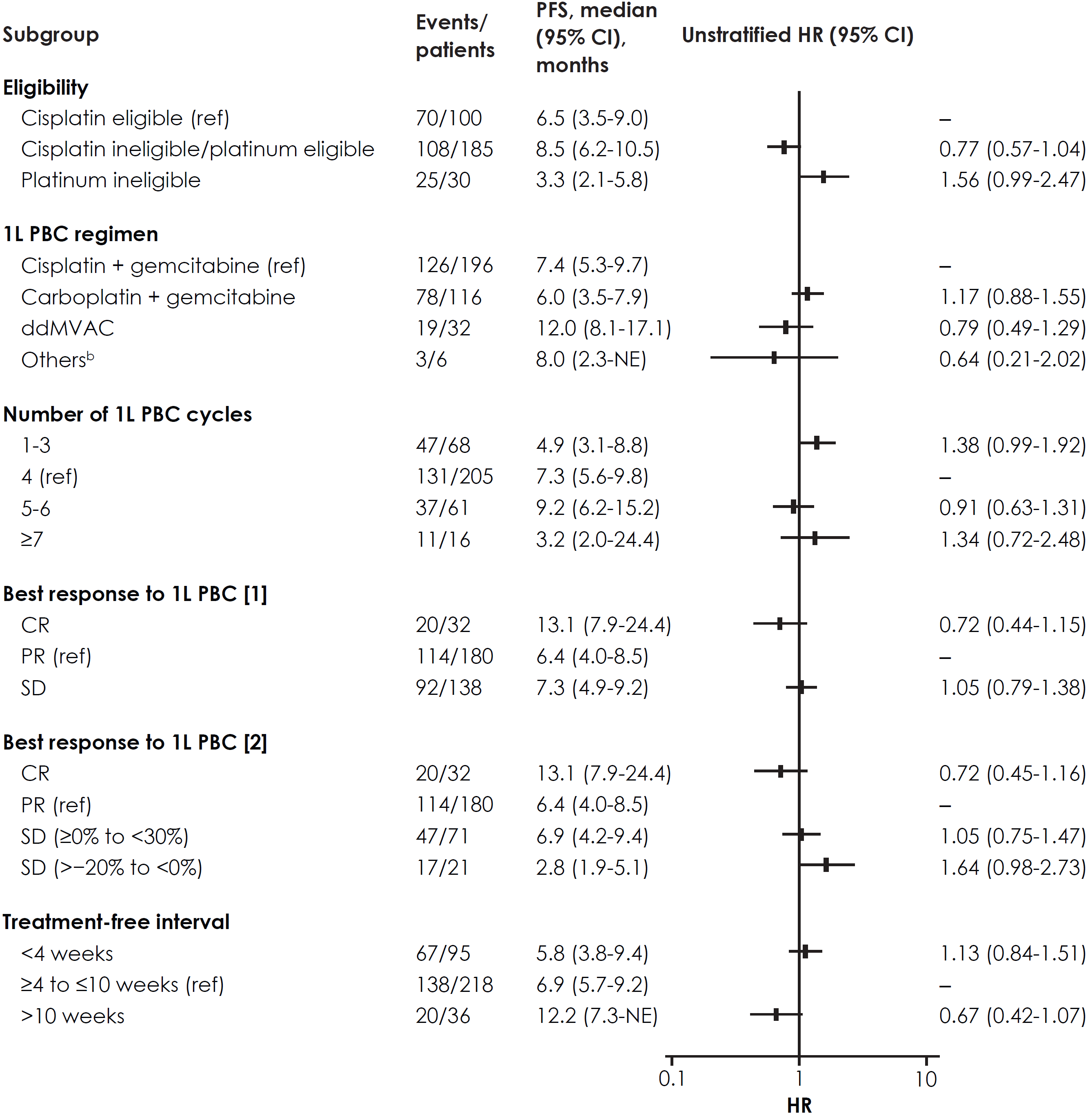


1L, first line; CR, complete response; ddMVAC, dose-dense methotrexate, vinblastine, doxorubicin, and cisplatin; HR, hazard ratio; NE, not estimable; NR, not reached; PBC, platinum-based chemotherapy; PR, partial response; PFS, progression-free survival; ref, reference; SD, stable disease.

^a^Univariate analyses are exploratory and were not adjusted for multiplicity.

^b^Other 1L PBC regimens included nedaplatin + gemcitabine (n=4), cisplatin + etoposide (n=1), and carboplatin + etoposide (n=1).

**Supplementary Figure 2. OS (A) and PFS (B) from start of avelumab by best response to 1L PBC.**

**A**


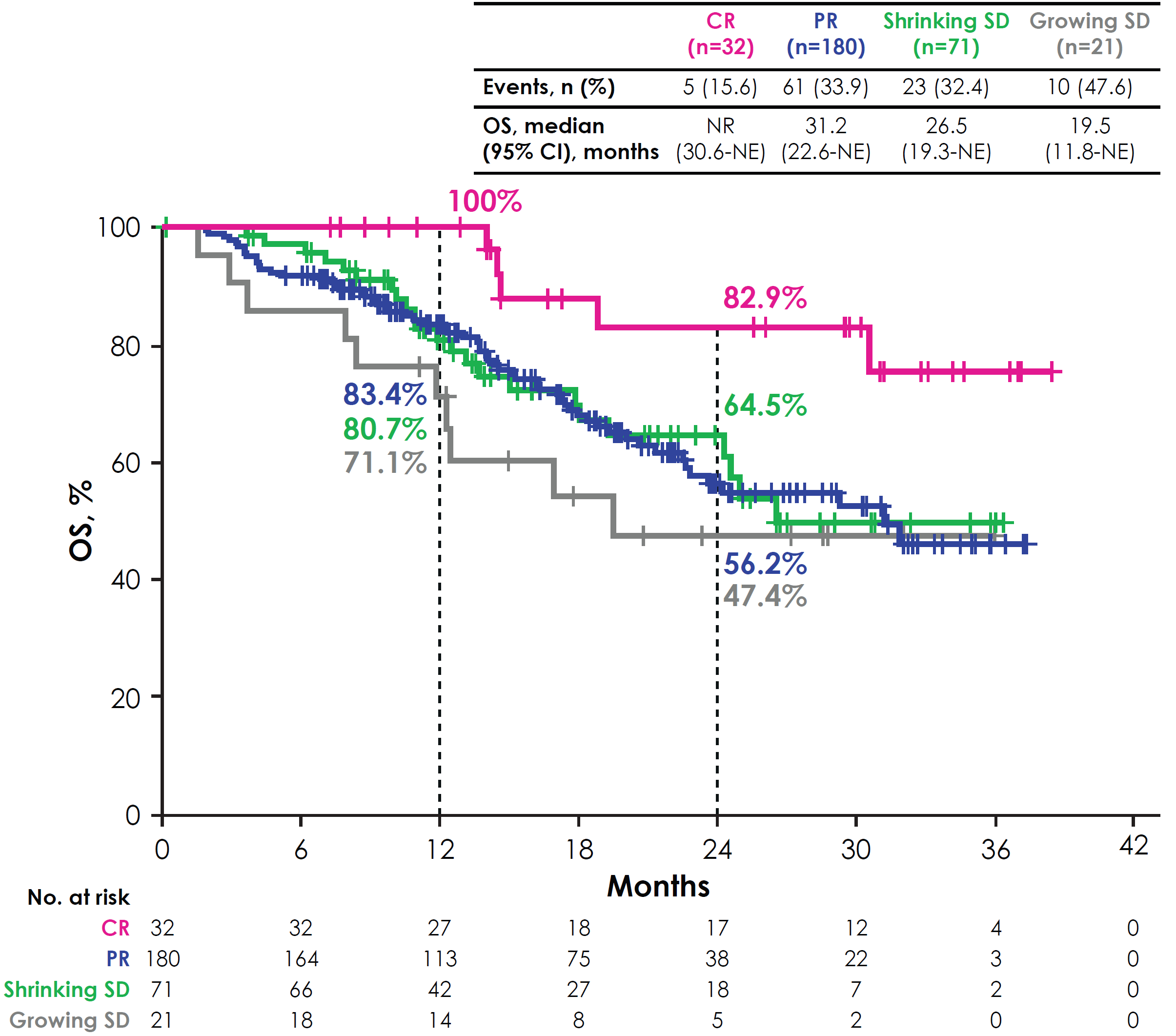


**B**


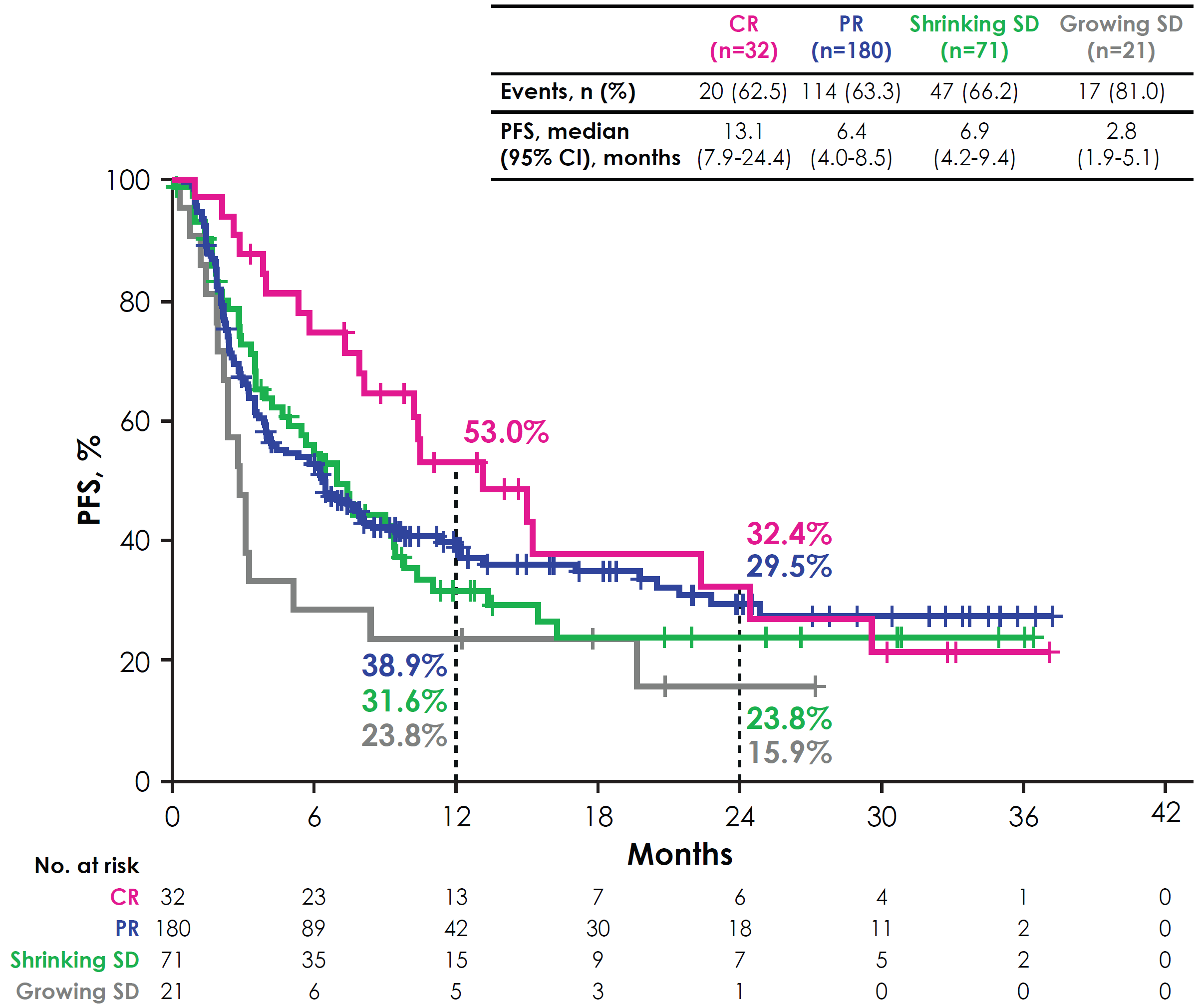

Exploratory stratification of RECIST SD based on its proposed heterogeneous nature and previous reports that subsets of patients have differential outcomes to immunotherapy based on the depth and direction of tumor change. Shrinking SD was defined as a ≥0% and <30% decrease in tumor size, and growing SD was defined as a >0% and <20% increase in tumor size.

1L, first line; CR, complete response; NE, not estimable; NR, not reached; OS, overall survival; PBC, platinum-based chemotherapy; PFS, progression-free survival; PR, partial response; SD, stable disease.
